# Maturing heart muscle cells: Mechanisms and transcriptomic insights

**DOI:** 10.1016/j.semcdb.2021.04.019

**Published:** 2021-05-02

**Authors:** Sean A. Murphy, Elaine Zhelan Chen, Leslie Tung, Kenneth R. Boheler, Chulan Kwon

**Affiliations:** a Division of Cardiology, Department of Medicine, Johns Hopkins University School of Medicine, Baltimore, MD 21205, USA; b Department of Biomedical Engineering, Johns Hopkins University School of Medicine, Baltimore, MD 21205, USA; c Department of Cell Biology, Johns Hopkins University School of Medicine, Baltimore, MD 21205, USA; d Institute of Cell Engineering, Johns Hopkins University School of Medicine, Baltimore, MD 21205, USA; e Cellular and Molecular Medicine, Johns Hopkins University School of Medicine, Baltimore, MD 21205, USA

**Keywords:** Cardiomyocyte maturation, Pluripotent stem cells, Single-cell transcriptomics, Tissue engineering, Maturation metrics, Heart development

## Abstract

Cardiomyocyte (CM) maturation is the transformation of differentiated fetal CMs into adult CMs that involves changes in morphology, cell function and metabolism, and the transcriptome. This process is, however, incomplete and ultimately arrested in pluripotent stem cell-derived CMs (PSC-CMs) in culture, which hinders their broad biomedical application. For this reason, enormous efforts are currently being made with the goal of generating mature PSC-CMs. In this review, we summarize key aspects of maturation observed in native CMs and discuss recent findings on the factors and mechanisms that regulate the process. Particular emphasis is put on transcriptional regulation and single-cell RNA-sequencing analysis that has emerged as a key tool to study time-series gene regulation and to determine the maturation state. We then discuss different biomimetic strategies to enhance PSC-CM maturation and discuss their effects at the single cell transcriptomic and functional levels.

## 1. Introduction

Heart muscle disease, or cardiomyopathy, is a leading cause of mortality and morbidity worldwide [1]. However, it is difficult to obtain cardiomyocytes (CMs) from patients, and they are often not amenable to functional analysis, making it difficult to study heart disease in vitro. While non-human mammalian model systems have been instrumental in determining some genetic causes of human cardiac disease, profound physiological and developmental differences among species (e.g., electrophysiology, contractility, metabolism, & immunology) confound data interpretation. As an alternative, pluripotent stem cells (PSCs) offer the ability to generate cells of any type in the body on a large scale. Therefore, they are considered to have tremendous potential for disease modeling, drug and cardiotoxicity testing, and regenerative medicine.

Human PSCs (hPSCs) have been successfully generated from a variety of sources. Conventionally, embryonic stem cells (ESCs) are derived from the blastocyst-stage inner cell mass. These cells are self-renewing and are pluripotent with the ability to differentiate in vitro into most cell types, with the exception of placental cells [2]. Embryonic germ cells (EGCs) derived from primordial germ cells are phenotypically similar to ESCs, but are often plagued by epigenetic changes, which in mouse affect their ability to generate viable embryos. Ethically, concerns associated with human embryonic tissue use preclude the routine generation of these PSCs for experimentation. Fortuitously, PSCs can also be generated by somatic cell nuclear transfer via transplantation of an adult nucleus into an oocyte [2] or by reprogramming somatic cells with a combination of transcription factors (e.g., OCT4, SOX2, KLF4, c-MYC) and/or microRNAs [3,4,5]. These experimentally generated PSCs, named induced PSCs (iPSCs), avoid ethical concerns associated with the use of human embryonic tissues and are used widely in the field [4].

Viable PSC lines can differentiate into all three germ layers and their derivatives, including CMs. This is largely owing to advances in our understanding of cardiac developmental biology, which has been instrumental for producing CMs from PSCs [6,7]. These advances have led to the development of methods that efficiently promote the differentiation of PSCs into PSC-CMs that yield more than 90% beating CMs [8] as well as chamber-restricted cardiac cells [9,10]. This has enabled us to create a near limitless source of PSC-CMs for broad biomedical applications [122]. As an example, hPSC-CMs that retain genetic mutations have been used to model successfully cardiomyopathies and channelopathies in vitro [11,12,13]. These cells can also be applied to drug discovery for personalized therapeutics, [14] and to repair damaged hearts either through direct injection or to create tissue patches with other cell types [15,16].

While methodological advances have significantly improved CM specification, yields, and purity [123–126], the practical use of CMs for these applications are plagued by several problems, including immaturity and interline variabilities intrinsic to PSC lines. In particular, PSC-CMs experience “maturation arrest” and resemble fetal or perinatal CMs, even following extended culture [17,18,81,117,118,119]. This undermines hPSC-CMs’ capability to fully recapitulate in vivo CM responses for disease modeling, drug screening, toxicity testing, and drug development. To tackle these problems, it is crucial to understand the biological process and mechanisms of CM maturation in vivo and define how these recapitulate normal developmental processes.

## 2. In vivo CM maturation

Human heart development bears significant resemblance to other mammals [19]. Small mammalian animal models, including mouse, rat, and rabbit, have been used to study heart development and heart diseases over the past two decades, filling in for the scarcely available primary human CMs [20]. Among these models, mouse models are the ones most frequently used for their high breeding rate, short lifespan, high genetic similarity with humans, and high capability for genetic modification [20]. However, mouse heart is different from human in beat rate, action potential duration, contractile profile and isoform expression with varying degrees, which need to be noted [20,21,22]. Human embryonic/fetal and adult CMs also display distinctly different phenotypes. Embryonic and fetal CMs are small with an amorphous cell structure. These cells spontaneously contract, have rudimentary calcium cycling mechanisms, possess nascent sarcomere structures, express “fetal proteins” and rely predominantly on glucose as their primary fuel sources. Adult CMs by comparison are large, rod shaped structures with well-developed calcium cycling mechanisms and well-defined sarcomere structures, are metabolically dependent on fatty acid utilization and express both adult- and chamber-specific protein isoforms. These differences can serve as a quick check list for discerning the relative maturity of PSC-CMs generated in vitro. Here, we will discuss the main characteristics of CM maturation in human and animal models, and highlight differences among these systems. We will use this information to establish a framework to better assess hPSC-CM maturation in terms of morphology, function, metabolism and transcriptomics. However, we will focus our discussion on ventricular CM maturation as most PSC-CM studies have centered on this population.

### 2.1. Morphology

Human CMs undergo significant changes in size and shape during the course of maturation. In human, adult CMs are large, elongated, and polyploid while immature CMs appear small, cobblestone-like, and euploid. Human CMs undergo developmental hypertrophy during the first 20 years of life, while in mice, the equivalent process is much shorter and is about 3 months, with a drastic increase of CM volume from postnatal day 5 to day 14 (P5-P14) [21,23]. Specifically, mouse CMs start to take on the signature elongated rod-shape and become binucleated in early post-natal stage from P4 to P6 [23,24,25]. The exact timepoints for the corresponding processes in human heart are not as well-documented as in animal models. Past studies described that binucleation was observed at week 32 of gestation and in approximately 94.3% of CMs in a human infant heart which is less than 1 day old [26, 27]. Furthermore, the elongated rod-shape was observed in a four-week-old human infant heart [28]. These results indicate that key morphological changes take place in the perinatal to postnatal stage in human. Structure-wise, both human and mouse adult CMs form highly organized sarcomere structures with aligned myofilaments along the longitudinal axis and intercalated discs joining the polar ends of neighboring CMs [23,24,25]. In murine heart, immature CMs exhibit less organized sarcomere structures with unaligned myofilament and non-restricted cell-cell contact located circumferentially on the cell membrane [29]. Myofilament alignment increases during early embryonic stage from embryonic day 9.5 (E9.5) and become almost fully aligned by early postnatal day 4 (P4) [23,29]. Meanwhile, adherens junction proteins start localizing to the polar ends of CMs at embryonic day 18.5 (E18.5) and become highly restricted to polar ends at birth, indicating the initial formation of intercalated discs [29]. In addition, immature CMs lack T-tubules, the invagination of the sarcolemma with localization of proteins involved in excitation-contraction coupling [30]. T-tubules, developing relatively later in the CM maturation process, are observed one week after birth and are fully developed at one month after birth in rat [25,30]. In human, t-tubules were observed at 32 weeks of gestation, situating the t-tubule development in human heart in the late fetal stage [26]. Moreover, the initial coupling between t-tubule and the sarcoplasmic reticulum (SR) was also observed at 32 weeks of gestation, showing the development of spatial organization of excitation-contraction coupling apparatus in the late fetal stage.

### 2.2. Contraction and calcium handling

Cardiac muscle contraction is the primary power source for arterial blood circulation and an essential function of heart. Contraction is fulfilled by a series of events called excitation-contraction coupling, where the initial depolarization leads to a small influx of Ca^2+^ from the L-type calcium channels and subsequently leads to a large efflux of Ca^2+^ from the sarcoplasmic reticulum (SR) through the ryanodine receptors (RyRs) into the cytoplasm [31,32]. Next, Ca^2+^ binds to troponin C leading to a conformational change, which initiates the sliding of thick and thin filaments and results in cell shortening and contraction at the organ level to eject blood out of the heart. During relaxation, RyRs close, and cytoplasmic Ca^2+^ is recycled back to the SR through sarco/endoplasmic reticulum Ca^2+^-ATPase (SERCA) or pumped out of the cell through the sarcolemmal Ca^2+^-ATPase and Na^+^/Ca^2+^ exchanger (NCX). To produce robust cyclic contractions in the heart, electrical excitation, calcium handling, myofilament organization, and energy supply through metabolism all play crucial roles. During development, the heart is the first organ to form in an embryo, and it starts contracting at approximately the third week during gestation in human [33,34]. In murine heart, contraction starts at around E8; however, cardiac crescent and spontaneous asynchronous Ca^2+^-oscillations (SACO) are observed at E7.75, suggesting the early onset of calcium cycling prior to CM contraction [33]. Knockout of the cardiac RyR (*RYR2*) in mice results in embryonic lethality [35], while inhibition of NCX1 in mouse embryo has been shown to dysregulate Ca^2+^ handling and disrupt CM differentiation, indicating that Ca^2+^ plays a crucial role in heart development and contractility [33].

A study by Hwang et al. compared three hPSC-CM cell lines generated in three different labs, using a monolayer-based cardiac differentiation protocol, to mature rabbit and mouse CMs [36]. This study showed that CMs from all three cell lines exhibit cytoplasmic Ca^2+^ buffering, L-type Ca^2+^ current, twitch Ca^2+^ transients, and caffeine-releasable SR Ca^2+^ stores comparable to those in rabbit CMs. The twitch Ca^2+^ transients and SR Ca^2+^ stores also increased in culture from day 15 to day 21 after induction. However, this study showed significantly slower transport by SERCA and NCX in hPSC-CMs relative to mature rabbit and mouse CMs. This is likely due to the immaturity of PSC-CMs, specifically, the poor development of T-tubule and SR [32,37]. To note, species differences can also affect the results in this study. T-tubules are organized as a network throughout the cell and bring L-type Ca^2+^ channels, RyRs, and the SR in close proximity, enabling a sensitive response of SR release of Ca^2+^ to the initial Ca^2+^influx through the L-type Ca^2+^ channel, as well as a synchronous calcium rise within the cell. Lack of T-tubules in immature hPSC-CMs can lead to inefficient activation and slower calcium handling kinetics [37,38]. Apart from the lack of T-tubules, hPSC-CMs were found to have functioning but immature SR with low expression of SERCA2a and NCX1 compared to their hESC-CM counterpart [38].

### 2.3. Electrophysiology

The electrical activity of the heart is critical for normal, regulated cardiac contractile activity. Abnormalities in cardiac conduction, repolarization, and heart rate can lead to cardiac arrhythmias that can result in cardiac dysfunction or arrest during development and in adults. Immature hPSC-CMs have unique expression profiles of proteins involved in ion transport compared to adult CMs, which can result in poor recapitulation of electrophysiological response in hPSC-CM applications including drug screening and toxicity testing [39].

hPSC-CMs commonly beat spontaneously. Several factors can lead to this trait. One of them is the low density of inward-rectifying K+ channels (I_K1_ current), specifically the lack of key building blocks Kir2.1 and Kir2.2 proteins. The reduced level of KCNJ2 expression and subsequently the reduced I_K1_ leads to a less negative resting membrane potential in hPSC-CMs, usually between −50 and −60 mV compared to around −80 mV in adult human CMs [22,40,41]. The less negative resting membrane potential makes cells more easily excitable and thus susceptible to spontaneous beating as seen in immature CMs [41]. Previously, the pacemaker current or funny current (I_f_) has been suspected to cause spontaneous beating in hPSC-CMs. However, even with upregulated expression of pacemaker channels encoded by *HCN* family genes, inhibition of I_f_ by ivabradine had no effect on the spontaneous beating of hPSC-CMs [41]. Recent studies have shown that a Ca^2+^-clock mechanism can be responsible for the automatic activity [41,42]. Spontaneous release of Ca^2+^ from the SR via RyR can elevate cytosolic Ca^2+^ concentration, activate the forward-mode of NCX, and produce an inward depolarizing current that initiates an action potential [41].

Sodium-Calcium exchanger (NCX) plays a key role in Ca^2+^ extrusion and intracellular calcium homeostasis [43]. In previously mentioned studies for hPSC-CMs, Lee et al. observed decreased NCX1 expression level and Hwang et al. reported slower Ca^2+^ transport [36,38]. Furthermore, the Ca^2+^ transport rate via NCX increased with prolonged culture time from day 15 to day 21 post induction [36]. In vivo, NCX expression level changes throughout the lifespan. In rat, NCX expression level is high in both fetal and neonatal stages, peaks at embryonic day 19 (E19), and gradually decreases to adult level after birth [13,43].

In addition to the aforementioned electrophysiological traits observed in hPSC-CMs, Cordeiro et al. reported robust transient outward current (I_to_) but with slow recovery kinetics in hPSC-CMs. The study also showed an increase in K_v_1.4 expression and a large decrease in KCHIP2 expression compared to human right ventricular tissues [44]. Zhao et al. reported an increase in volume-regulated chloride channel (LRRC8A, LRRC8B, LRRC8E) expression levels in hPSC-CMs at longer (50–60 days) compared to shorter (30–40 days) differentiation times [45].

Immature hPSC-CMs also exhibit differences in cardiac sodium (Na^+^) channels compared to adult CMs. Goodrow Jr *et al.* reported that the action potential recordings of hPSC-CM monolayer show a phase 4 depolarization and a low upstroke velocity, indicating low level of I_Na_ [46]. This can result from the expression of the fetal isoform of sodium channel Na_v_1.5 in immature hPSC-CMs [47]. Sodium channel Na_v_1.5 is responsible for mediating I_Na_ and is encoded by *SCN5A* [47]. In mice, the development of sodium channels initiates in the embryonic stage and continues during postnatal development [48,49]. From the early embryonic stage (10.5–12.5 days post coitum) to the late embryonic stage (16.5–18.5 days post coitum) in mice, both Na^+^ current density and recovery rate from inactivation increase, demonstrating Na^+^ channel development during embryonic development [48]. There is also an upregulation of Na^+^ channel subunits Na_v_1.4, Na_v_1.5, Na_v_1.6, Na_v_1, Na_v_2, and Na_v_3 in late embryonic stage in mice ventricles [48]. Furthermore, gene ontology studies showed that the expression of ion transport genes are upregulated in neonatal mouse heart during the first three weeks after birth, showing continued development of ion channels in the post-natal stage in CM maturation [49].

The cell-to-cell electrical coupling via gap junctions, located in the intercalated discs, is also critical for proper electrophysiological function [50]. The intercalated discs are located on the longitudinal ends of adult CMs. In hPSC-CMs, however, the intercalated disc components, including connexin 43, N-cadherin-mediated adherens junctions, Na_v_1.5, and desmosomes, are dispersed around the cell perimeter [50].

### 2.4. Metabolism

The heart is highly dependent on oxidative energy generated in mitochondria for excitation-contraction-relaxation coupling. In fact, over 35% of the total cell volume of adult CMs is occupied by mitochondria that spatially are aligned with myofibrillar proteins [51,52]. Adult CMs thus rely on fatty acid β-oxidation, respiratory electron chain and oxidative phosphorylation for most of its energy needs. Meanwhile, immature CMs rely on glycolysis as the main source of energy, and not mitochondrial oxidative metabolism [53]. Developmentally, CMs switch metabolic substrate from glucose to fatty acid shortly after birth, and the mitochondria mass increases rapidly to meet the growing demand of energy [34,54,53]. Glycolytic metabolism seems to maintain the proliferative state of CMs while increased mitochondrial oxidative metabolism seems to drive CMs to a more terminally differentiated stage [55, 56]. Therefore, the metabolic switch is suspected to have impacts on CM maturation.

hPSC-CMs, by comparison, have low numbers of mitochondria that are spatially located in the peri-nuclear regions of a cell. These cells are highly dependent on glucose as their main energy source. Early in vitro differentiated CMs also are resistant to hypoxia. With time of in vitro differentiation, mitochondrial numbers increase, and these organelles re-orient to be associated with myofibrillar proteins. Moreover, PSC-CMs with enhanced mitochondrial mass show improved fatty acid uptake, improved β-oxidation, enhanced ATP production and show enhanced sensitivity to oxidative stimuli [57]. Several studies looked into PSC-CM maturation in the presence of fatty acid [58,59,60,61]. These studies reported enhanced cell morphology, stronger twitch force, enhanced calcium handling, faster action potential upstroke velocity and increased oxidative capacity, showing that the metabolic switch is a key player in CM maturation.

### 2.5. Gene expression

The transcriptomic profile of CMs evolves during the course of maturation. To date, RNA and protein distinctions between immature CMs and adult CMs have been partially identified and utilized to evaluate the maturity of differentiated hPSC-CMs. At the gene level, isoform switching is observed in several proteins during CM maturation. Connexin 40 (Cx40) and Cx43 are both gap junction proteins and enable action potential propagation between CMs. Cx40 is widely expressed in the ventricles at E11 in mouse heart and is restrictively expressed in the ventricular conduction system by E14. Cx43 becomes uniformly expressed in the ventricle of adult mice [62,63,64,65]. Isoform switching is also observed in the sarcomeres. Myosin heavy chain (MHCα), encoded by *MYH6*, is predominantly expressed throughout rodent fetal heart and in human fetal atrium. Low levels of MHCα are present in human fetal ventricle [120]. Between week 7 to week 12 in gestation in human fetal heart, the level of MHCα decrease further while the level of MHCβ, encoded by *MYH7*, becomes higher [66,67]. Furthermore, Gorza et al. reported that MHCα is mainly found in the atrium of adult human hearts and is less abundant in ventricles. Similarly, MHCβ is prevalent in ventricles and is poorly abundant in atrium [68]. Myosin regulatory light chain is mainly present in the form of MLC2a in the ventricles in early fetal mice embryo and later undergoes an isoform switch to be MLC2v [69]. Similarly, slow skeletal troponin I (ssTnI), encoded by *TNNI1*, is predominantly expressed in fetal CMs. During the perinatal period in human heart, isoform switching occurs as cardiac troponin I (cTnI), encoded by *TNNI3*, takes over [70]. Bedada et al. also showed the positive correlation between the cTnI to ssTnI protein isoform ratio to the maturation status of CMs [71]. In addition, titin, which impacts the muscle stiffness of the myocardium, also undergoes an isoform switch. Titin is mainly in the form of *N2BA* in the fetal state and is later switched to a stiffer isoform *N2B* [72].

The transcriptomic profiles of hPSC-CMs defined by microarrays and RNA-seq analyses are distinct. While some of the early studies were limited due to an inability to purify in vitro derived CMs, microarray analyses demonstrated that PSC-CMs have an abundance of transcripts implicated in developmental processes and in cellular processes involving cell migration, focal adhesion, ion transport, growth factor signaling and cell survival [73,74,121]. For example, Ca^2+^ handling proteins, including RYR2 and SERCA2 and ventricular ion channel KCNJ2, are all upregulated with time of in vitro differentiation [22]. Other genes involved in cell communication, signal transduction and host defense responses that are up-regulated in fetal and adult CMs are not, however, prevalent in hPSC-CMs [73]. Two studies also employed the transcriptomic datasets to stage PSC-CMs relative to human cardiac samples. Poon et al. reported that the transcriptomic expression of PSC-derived ventricular CMs (vCM) grouped better with human fetal-vCMs than to either hESCs or adult ventricular CMs [121]. Van den Berg et al. subsequently demonstrated that the gene expression profile of PSC-CMs had a gene expression profile similar to that from a first trimester fetal heart, thus confirming that hPSC-CMs are immature [75]. Apart from the aforementioned isoform switches during the course of CM maturation, additional global differences in gene expression level are worth noting. Cell cycle gene transcripts are altered in both rodent and human CMs, and these changes are associated with a decrease and then loss of cell proliferative capacity during heart development [76]. More specifically, cell cycle genes *CDK1, CCNB1*, and *AURKB* are downregulated during CM maturation. Glycolysis-associated *HK1* is also downregulated, while peroxisome proliferator activated receptor (*PPARA*), a regulator of fatty-acid metabolism, is upregulated. A more extensive list of gene expression changes can be found by a recent review by Guo and Pu [22].

In summary, hPSC-CMs differentiated using current protocols are largely immature, and there exists a large distinction between immature hPSC-CMs and adult CMs. The transition from immature to mature is observed molecularly, structurally, functionally, and metabolically, and these changes occur across individually different timelines among CMs. The dynamics of maturation thus are a reflection of the complexity of CM development, and no single parameter can be employed to define “maturation.” To better define the process of CM maturation and how it relates to in vivo CM development, it is crucial to develop reproducible metrics to stage the maturity of hPSC-CMs. Once these metrics have been defined, the information can be used to guide in-house generation of matured hPSC-CMs for research and application purposes.

## 3. Gene regulatory networks and pathways influencing CM maturation and transcriptomic studies

### 3.1. Single-cell RNA-sequencing (scRNA-seq) to study maturation

With the goal of understanding CM maturation in vivo, Uosaki et al. sought to use the vast amounts of microarray data available to build a meta-analysis of developing CMs from early embryonic to adult timepoints [17]. Gene expression trends were tracked along with predicted upstream regulators among stages. Such analyses led to identification of activated pathways including PPAR and Interferon regulatory factor (IRF) along with inactivated pathways of Transforming growth factor-β (TGF-β) and Hedgehog. While this study contributed to an understanding of overall gene expression changes and to the identification of dysregulated pathways, bulk analysis has limitations in determining cellular heterogeneity and biological trajectories. With the rise of scRNA-seq technology, the maturation process is actively being investigated at single cell resolution. This allows analyses to track heterogeneity and to assess specific cell types throughout the course of development or differentiation. In addition, scRNA-seq can focus on CMs only and track crosstalk among multiple cell types in the heart [77].

**Fig. 1.**
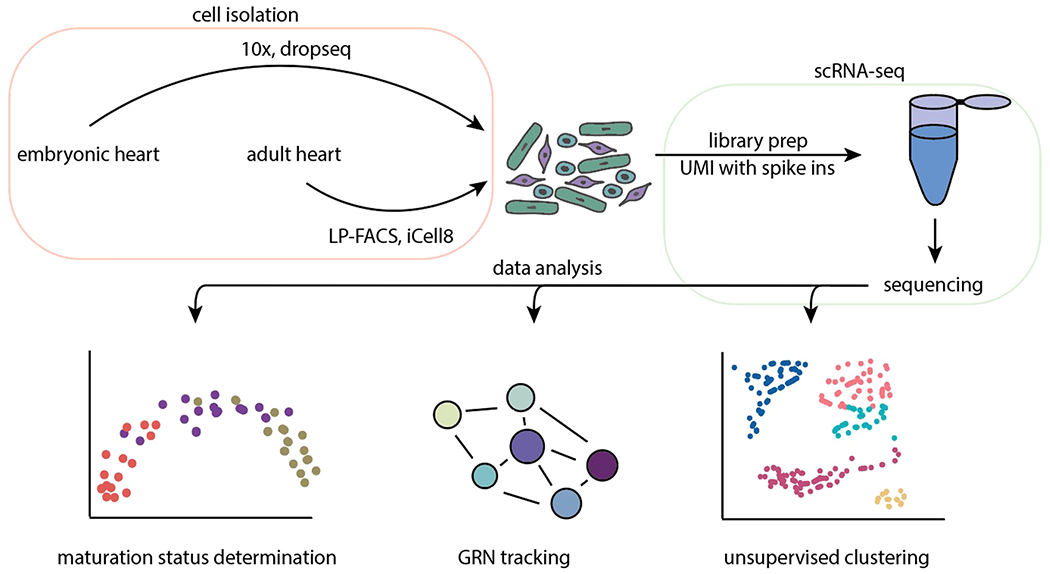
Overview of single-cell RNA-seq pipeline and downstream data analysis. Isolation of healthy cells from dissociated hearts depends on the timepoint as postnatal and adult CMs require LP-FACS or the iCell8, while drop-seq and 10X can be used for embryonic cells. Libraries are then prepared, preferably using UMIs and spike-ins to improve normalization. Following sequencing, downstream analyses include trajectory-based determination of maturation status, GRN reconstruction, and unsupervised clustering to group cell types. Acronyms: LP-FACs, large particle fluorescence-activated cell sorting; UMI, unique molecular identifier; scRNA-seq, single cell RNA-sequencing; GRN, gene regulatory network.

Despite the promise of scRNA-seq, a key technical hurdle remains in analyzing postnatal CMs. Previous studies were limited to cell picking and single nucleus isolation [78]. Single nucleus RNA-seq is ill suited for CM transcriptomic studies as it does not capture mitochondrial reads, and nuclei contain less than 10% of mRNA [79]. 10X Genomics (company based in Pleasanton, CA) offers accessible and relatively inexpensive sequencing for embryonic timepoints, but this is more conducive to identifying cell type rather than tracking gene expression trends, as most reads from a CM are associated with contractile or mitochondrial genes [79]. For example, DeLaughter et al. sequenced embryonic to P21 postnatal hearts using drop-seq [80], however, postnatal time points after P0 included very few cells. Most of these cells did not pass quality control cutoffs, probably because of difficulties in sorting CMs using conventional fluorescence-activated cell sorting (FACS). The relatively large size of post-natal CMs leads to shearing and damage. Only recently have iCELL8 (Takara Bio 640188) and large particle FACS (LP-FACS) emerged as viable methods (Fig. 1) for sorting healthy adult CMs [81, 127]. Therefore, at present, LP-FACS/iCELL8 isolation with deep sequencing (>500k reads per cell) may be the best practice for tracking CM gene expression. Indeed, Murphy et al. generated a high-quality trajectory of postnatal CMs with LP-FACS and successfully inferred key transcriptional regulators in CM maturation [82].

In vitro PSC-CM maturation has been studied using single cell and bulk RNA-seq. Using 10X Genomics sequencing of PSC-CMs undergoing differentiation, Friedman et al. identified markers of different progenitor and cell type populations [83]. Another study used bulk RNA-seq to assess the effect of long-term culture of PSC-CMs up to day (d) 200 and found that PKA/proteasome and HSP90-signaling pathways upregulate mitochondrial energy production over time [84]. A major drawback is that d200 PSC-CMs are still immature, and the effect was not validated in vivo. Nevertheless, scRNA-seq is emerging as a prominent method in both predicting changes in maturation and assessing the impact of key regulators on postnatal development.

### 3.2. Pathways required for maturation

Currently, the best systems to study in vivo pathways of maturation rely on mouse models of post-natal heart development amenable to genetic modification. Using an *MYH7-YFP* mouse line as a reporter of maturation in a CRISPR/Cas9 knockout screen, Vandusen et al. found RNF20/40 to be key epigenetic regulators required for maturation [85]. Knockdown of estrogen-related receptor (ERR) isoforms ERRα and ERRβ postnatally showed that these regulators are critical for CM maturation [86]. ERR proteins are nuclear receptors that regulate mitochondrial biogenesis, and in mice with high levels of ERR knockdown, cardiomyopathies developed. Using ChIP-seq, Sakamoto et al. found that ERR also suppresses fibroblast genes and upregulates contractile and ion channel genes [86]. HOXB13 is a cofactor of MEIS1 and cooperates to regulate CM maturation to initiate cell cycle arrest [87]. When these factors are knocked out, adult hearts show dysfunction from sarcomere disorganization and CM proliferation.

External mechanical forces can also regulate CM maturation and be translated into transcriptional changes through mechanosensing [88, 128]. Vinculin is key to maturation as it recruits slingshot protein phosphatase (SSH1) and actin depolymerizing factor cofilin to regulate myofilament maturation. Maturation can be derailed by knocking out proteolysis machinery. In this case, knockout of E3 ubiquitin ligase ASB2 is required for CM maturation in zebrafish in a transplanted model [89]. ASB2 degrades transcription factor TCF3, which prevents maturation. In a study of the role of TLR3 in cardiac reprogramming, Hodgkinson et al. found that TLR3 activates NFκB, as TLR3 or NFκB knockdown prevents maturation [90]. However, this study primarily used qPCR measurements of gene expression of contractile genes including Myh6, Actn2, and Tnni3 to determine maturation state, but did not quantify contractility, calcium handling, or morphology.

Since CM dysfunction can lead to global heart problems that activate feedback loops, mosaic knockouts of function have been developed in mouse to identify pathways required for CM maturation. The most prominent system for mosaic knockouts is CASAAV, which uses an adeno-associated virus to express sgRNAs and Cre Recombinase under a cardiac promoter in loxP-stop-loxP-Cas9-GFP mice [91]. It was validated with a test of t-tubule disruption using a cardiac-specific Junctophilin-2 mosaic knockout. Using a mini-screen of 9 factors in CM development, they found that postnatal knockout of Serum Response Factor (*SRF*) prevents morphological and functional maturation [92]. In a follow up study, it was determined that *SRF* knockout has a similar phenotype to the *ACTN2* knockout phenotype which can be recreated by *MRTFA* mutant [93]. From these studies, we can conclude that CM maturation is not controlled by the same factors involved in CM differentiation. Instead, maturation appears to be driven by a complex regulatory network rather than a single master regulator.

### 3.3. Pathways that promote PSC-CM maturation

**Fig. 2.**
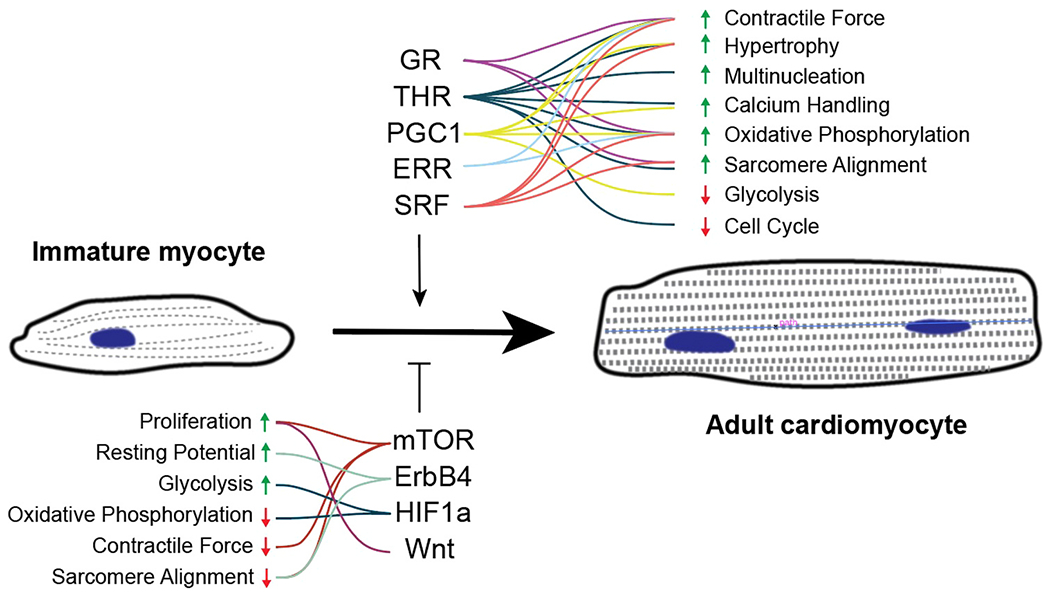
Pathways affecting CM maturation. Summary of factors show to activate or suppress individual aspects of maturation. Factors that promote maturation and those that block maturation are shown. Acronyms: GR, glucocorticoid receptor; THR, rhryoid hormone receptor; PGC1, Peroxisome Proliferator-activated receptor-γ coactivator 1; ERR, estrogen-related receptor; SRF, serum response factor; mTOR, mechanistic target of rapamycin; ErbB4, epidermal growth factor receptor; HIF1a, hypoxia induced factor 1α; Wnt, Wnt singaling pathway.

A number of pathways have been shown to improve in vitro PSC-CM maturation when activated (Fig. 2). Triiodothyronine (T3) binds to the thyroid hormone receptor to promote maturation of PSC-CMs with more force production and better sarcomere organization [94,95]. It also leads to mitochondrial maturation, as the maximal oxygen consumption is increased, although not with an increase in mitochondrial genome number. The thyroid hormone receptor has been proposed as a key factor in the loss of cardiac regeneration in mammalian species. The glucocorticoid receptor is another nuclear receptor that promotes maturation when activated by hydrocortisone or dexamethasone [96, 97]. When T3 and dexamethasone are combined in PSC-CMs cultured on a matrigel mattress, functional t-tubules form [98]. Since fatty acid treatment promotes maturation and PGC1/PPAR was a top predicted transcriptional regulator of maturation, the role of PGC1α in postnatal CM maturation has been investigated [82]. Not only is PGC1 required for morphological, transcriptomic, and functional maturation, but activation of PGC1 and PPAR through Pyrroloquinoline quinone or WY14643, respectively, promotes PSC-CM maturation. Activation of this pathway with ZLN005, another PGC1 activator, has similar results on PSC-CMs [99]. microRNAs affect maturation by degrading mRNA through the RNA-Induced Silencing Complex. Primary microRNA are trimmed by DICER and Drosha to form mature 21–22 bp microRNA. Knocking out DICER leads to dilated cardiomyopathy indicating that microRNAs are critical for heart function [100]. microRNA *Let-7* overexpression leads to CM hypertrophy, improved contractility, and better mitochondrial function, perhaps by downregulating the PI3K/AKT pathway and up-regulating fatty acid oxidation [101]. Lee et al. found a cocktail of four microRNAs including *miR-125b-5p, miR199a, miR-221*, and *miR-222* that lead to electrophysiologic and contractile maturation [102]. microRNA target analysis showed that all four bind and down-regulate ERBB4. HOPX was identified as a regulator of cellular hypertrophy in maturation [83]. Overexpression of this factor led to increased cell size, but other aspects of maturation were not determined.

### 3.4. Pathways that block PSC-CM maturation

PSC-CMs, in vitro, may have dysregulated signaling or gene expression that prevents them from maturing. Inhibition of the mTOR signaling pathway with Torin1, for example, increases the proportion of quiescent PSC-CMs and expression of maturation related genes [103]. Functionally, treatments with Torin1 improves contractile force, contraction dynamics, and mitochondrial function. Inhibiting mTOR leads to upregulation of *TP53* (p53), which drives cell cycle arrest. In a study of expanding PSC-CMs in vitro, Buikema et al. found that PSC-CMs matured with increased contractility and higher levels of maturation gene expression when GSK-3β was removed, indicating that this signaling molecule may block maturation [104]. In a study investigating why media containing high levels of glucose prevent maturation, Nakano et al. found that inhibition of HIFα or lactate dehydrogenase A leads to a switch from glycolysis to fatty acid oxidation in PSC-CMs [105]. This results in improved contractility and upregulation of maturation-related genes, and it suggests that HIF1α promotes glycolysis as the primary form of metabolism. Dysregulation of the gene expression of key maturation regulatory networks may also be important for CM maturation. As one example, *miR-200c* was identified by Poon et al. as a maturation inhibitor through bioinformatics analysis [106]. miR-200c targets *GATA4, SRF*, and *TBX5* for degradation in addition to the L-type calcium channel. The presence of miR-200c thus represses both maturation and differentiation, and dysregulation of its expression in vitro may affect the timing of CM maturation. Abnormal cell cycle progression is observed in *Alms1*-deficient PSC-CMs, suggesting that Alms1 may regulate terminal differentiation of CMs [133]. This is consistent with the finding that mutation of *ALMS1*, a causative gene for Alström syndrome, can lead to mitogenic cardiomyopathy in humans [133]. Further investigation would be needed to elucidate the pathway involving Alms1 that regulates cell cycle activity.

## 4. Maturation metrics

Maturation is a transformation that includes many changes in morphology, function, and epigenomic and transcriptome changes. This section will review maturation metrics to identify the best options for researchers seeking to assess CM maturation status. Since maturation is a complex biological event, a panel of indices are required to assess it. Qualitative methods involving protein staining have been used to assess sarcomere organization, cell morphology, and mitochondrial structures. Quantitative measurement of force is an excellent way of assessing contractility; however, comparing force measurements of PSC-CMs across labs has proved challenging [107,108,109]. Due to the difficulties in accurately assessing maturation, many investigators are now focusing on quantitative assessments of gene expression or transcript levels, either individually or as a pool of maturation markers.

Myomesin-2 (MYOM2) is a protein found in cardiac and skeletal tissues in the M-band, where it connects to titin. MYOM2 is differentially expressed during maturation and increases 8-fold from embryonic to adult timepoints, and it has been described as a reliable marker of maturation [110]. Taking advantage of this property, Chanthra et al. fluorescently tagged Myom2 with RFP to create an in vitro reporter of maturation. As expected, this tagged protein localized to the M-band, could be used to visualize sarcomere organization, and increased its expression level with maturation. Taking advantage of this reporter line, the authors used this system to assess the entire transcriptome as a function of maturation. For this analysis, the authors created MatStat (a software package) to perform a meta-analysis of microarray data of hearts from early embryonic to adult time points to create gene regulatory networks at each stage [17]. The gene expression data from a sample was then compared to the networks of multiple timepoints to identify which stage of maturation was closest to the sample. The results indicated that PSC-CMs cultured in vitro did *not* progress beyond a late embryonic stage of development.

While MatStat requires the input of a bulk sequencing or microarray sample, newer methods have begun to quantify the maturation state on the single cell level. DeLaughter and colleagues used drop-seq scRNA-seq of E9.5, E14.5, P0, and P21 mouse hearts. Principal component analysis (PCA) showed a temporal trend in expression [80]. They then used bulk RNA-seq data to determine the maturation state of PSC-CMs by mapping them to PCA component 2 using a subset of 997 genes that were expressed in 75% of myocytes. Their results showed that mouse ESC-derived cardiac progenitors could be staged to E9.5 of mouse development, while mES-CMs were most similar to CMs from E14.5. By using human orthologs, they were able to map hESC-CMs to the PCA component that captured maturation progression, finding that d20 hESC-CMs aligned with E14.5 and d365+ hESC-CMs corresponded to E18.5.

Trajectory inference (pseudotemporal ordering) is a computational method used to determine the pattern of a dynamic events experienced by cells in pseudotime based on scRNA-seq progressions among cell states. In a study of postnatal maturation, mouse CMs were collected every 7 days from P0 to P28 and then ordered using Monocle [82,111]. This pseudotime scoring was titled ‘maturation score’ and was used to determine the maturation state for PGC1α/β knockout CMs. At each timepoint examined, the authors found lower maturation scores for the knockout CMs relative to the controls, showing these cells were morphologically and functionally less mature. Since trajectories may change with the addition of cells, and transcriptomic comparisons are plagued by batch effects and differences in protocol, Kannan et al. sought to generate a universal system capable of defining myocyte maturation in pseudotime independent of sequencing protocol [81]. To achieve this goal, the authors formulated a novel concept based on gene distribution. To test this idea, they used Shannon entropy and demonstrated that entropy levels gradually decrease from fetal to adult CMs, supporting the concept of entropy to quantify CM maturation [81]. By correcting wrong reads, filtering low quality cells, and selecting abundant genes, they further showed that entropy score can serve as a cross-study, cross-species metric of CM maturation [81]. This makes entropy score a promising, unbiased metric to quantify the status and trajectory of PSC-CM maturation.

As an alternative to mRNA-based analysis, proteomic based approaches were used to define maturation [87]. Using hPSC-CMs, Nguyen et al. assessed protein levels as a function of maturation in both 2D and 3D culture and determined that MYOM1 and TMEM65 levels were markers of maturation. This approach also proved informative for analysis of post-translational modifications. Specifically, they demonstrated a decrease in the phosphorylation of α-tropomyosin in more mature relative to immature PSC-CMs. Multiple markers were validated by looking at proteins isolated from mouse hearts.

While these techniques for assessing maturation metrics require transcriptomics or proteomics, which can be expensive or challenging to undertake, the results are comparable among research groups and more standardized than the current practice of measuring many different aspects of maturation. Additional studies are however required to further validate this approach. First, studies should be undertaken to confirm directly that transcriptomically mature CMs are indeed functionally mature via sequencing and measurements of the functional parameters of CMs. This is important because both mature and immature CMs are present at later stages of in vitro differentiation, and consist of heterogenous populations of CMs [57,82]. One strength of this process is that it takes into account the expression of thousands of genes. Pseudotime metrics can also provide precise estimations of the maturation status. Second, direct comparisons between transcriptomic and proteomic datasets are challenging. This is because the transcriptome does not perfectly match the proteome. The use of both transcriptomics and proteomics may ultimately be necessary to quantify maturation; however, it is likely that routine application of only one of these approaches will be sufficient to stage the maturation state of PSC-CM.

## 5. New approaches and analysis of CM maturation at single-cell resolution

CM maturation is a gradual, complex process that is neither synchronized nor constant among cells present in the same culture [57,82]. To overcome this obstacle, the study of CM maturation at single-cell resolution allows researchers to track the developmental trajectory for specific cell types, at different times and under disparate culture conditions. Single-cell resolution analysis can also reveal cellular heterogeneity, which would likely be masked in bulk data analysis [112]. While there are in vivo protocols developed to mature hPSC-CMs [129–131], this section will be focused on four in vitro studies from recent efforts to study hPSC-CMs with different types of external cues to promote maturation and assays at single-cell resolution. Among the four studies described here, two used scRNA-seq to evaluate the transcriptional profile, one used single cell western blotting to assess protein profiles, and one used both single-cell optical electrophysiological analysis and scRNA-seq. A table is provided (Table 1) summarizing the main findings collected with the corresponding single-cell resolution assays. This section provides a glance at state-of-the-art single-cell resolution maturation studies of hPSC-CM.

Giacomelli et al. promoted hPSC-CM maturation using 3D microtissue (MT) co-cultured with hPSC-derived cardiac fibroblasts (CFs) and cardiac endothelial cells (CEs) [113]. They reported improved maturation outcomes in these co-cultures compared to ones without CFs. These outcomes included improved sarcomere structure, formation of T-tubule-like structures, as well as improved contraction, Ca^2+^ handling, action potential parameters, and mitochondrial respiration capacity. Single-cell RNA-seq analyses showed that CMs in the tri-co-culture microtissues clustered closely to adult human CMs, which led to further investigation into the tri-co-culture group. Single-cell and bulk RNA-seq data revealed that cyclic AMP (cAMP)/β-adrenergic and cell junction assembly pathways are upregulated in MTs with CFs but not in MTs with non-cardiac fibroblasts, suggesting the importance of CFs in CM maturation. The group also proposed that increased cAMP levels facilitated Cx43 gap junction assembly, which may account for the enhanced maturation observed with CFs and CEs. Support for this mechanism was revealed following sustained cAMP signaling through exogenous dbcAMP, which improved sarcomeric organization, and silencing of Cx43, which led to a reduction in sarcomere organization. The 3D microtissue construction method in this study could be valuable for future studies, given that it can generate disease models from patients with rare genetic disorder with just 5,000 cells to evaluate the role of non-CMs in pathology. In addition to CFs, sympathetic neurons were shown to improve CM contractility [132], suggesting the importance of non-CMs in CM maturation.”

Lam et al. used two separate tissue platforms to model pulmonary atresia with intact ventricular septum (PAIVS) in vitro comparing six hPSC-CM lines generated from three patients and three healthy individuals [114]. They used human cardiac anisotropic sheets (hCAS) to look at action potential propagation and human cardiac tissue strips (hCTS) to evaluate contractile functions. Single-cell RNA-seq analyses were performed on control and diseased cell lines cultured on both platforms. It is worth noticing that the immaturity of hPSC-CMs may not undermine the recapitulation of disease phenotypes in this case as PAIVS pathogenesis mainly occurs in the first trimester and hPSC-CMs resemble fetal CMs at this stage. The group observed reduction of contractility and prolonged contraction and relaxation kinetics in hCTS derived from diseased cell lines. Subsequently, scRNA-seq, RT-qPCR, and immunofluorescence analyses also showed downregulation of proteins associated with the contractile apparatus in hCTS of diseased cell lines. Based on their observation, Lam et al. proposed that the intrinsic contractile abnormalities seen in PAIVS hPSC-CMs models can explain the global contractile function defects seen in PAIVS patients. The single-cell resolution experimental outputs from hCAS and hCTS studies are shown in Table 1. The outputs from the comparison between control and diseased cell lines are not reflected in Table 1 as we want to focus on data associated to hPSC-CM maturation process in this review.

Biendarra-Tiegs et al. performed scRNA-seq and Arclight analysis on hPSC-CMs at both day 12 and day 40 of differentiation using a monolayer protocol and looked simultaneously at transcriptional and electrophysiological profiles [115]. Overall, scRNA-seq data showed increased transcriptional maturation from day 12 to day 40. Notably, the authors found that the cell sub-types (ventricle-like, atrial-like, nodal-like) determined by classical markers and functional electrophysiology were incoherent. More specifically, they reported that cells displaying an atrial-like or ventricular-like action potential morphology do not necessarily express atrial- or ventricular-restricted markers. They also reported the presence of cells of unknown identity that were neither atrial-like nor nodal-like. Their findings indicate that classical gene markers are insufficient for determining cell subtypes during in vitro CM differentiation, at least at the time points examined. Functional data like the electrophysiological profile provided in their study underscore the need for evaluating hPSC-CM maturation from various perspectives.

Jabart et al. conducted single-cell protein expression analysis on hPSC-CMs using Single-Cell Western (scWestern) blot to track protein expression heterogeneity in hPSC-CM cultures over time [116]. This first of its type scWestern system is a microfluidic chip with precast polyacrylamide gel consisting of 6400 microwells that can track up to 12 proteins per cell. The scWestern system can provide molecular weight sizing information, in addition to immune-binding information, which is suited for distinguishing protein isoforms and off-target antibody binding. In this study, the authors focused on subpopulations of cells expressing only MLC2A, only MLC2V, or both, where MLC2A was used as a marker for atrial CMs and a relatively immature state, while MLC2V served as a marker for ventricular CMs. They reported that the largest increase in MLC2V expression occurs in subpopulations co-expressing MLC2V and MLC2A. Meanwhile, MLC2A expression increased marginally in this subpopulation. Furthermore, the percentage of cell co-expressing MLC2V and MLC2A increases over time as the percentage of cells expressing only MLC2A decreases. The authors suggested that these results could reflect the process of myofibril development between these cell populations, where MLC2A expressing cells transition to express MLC2V and MLC2A simultaneously and become more ventriclar-like. In addition, the researchers performed scWestern on hPSC-CMs generated from CRISPR/Cas9-edited iPSC lines with removal of *NR2F2, TBX5*, or *HEY2* transcription factor exons to study the mechanisms underlying increased expression of MLC2V. They found decreased expression of MLC2V in both *TBX5*- and *HEY2*-deficient lines but not in the *NR2F2*-deficient line. This result is exclusive to MLC2V and MLC2A co-expressing subpopulations, suggesting that the expression of MLC2V in MLC2V subpopulations lacking MLC2A may be governed by other transcription factors.

The four studies mentioned above can serve as an epitome of recent hPSC-CM maturation studies that apply single-cell resolution analysis. From the studies by Giacomelli et al. and Lam et al., scRNA-seq data can supplement other assays and enhance the resolution of cell evaluation. Furthermore, scRNA-seq data and gene ontology (GO) analysis facilitate both studies in narrowing down targets for further investigating experimental findings. Single-cell level analysis also exposes heterogeneity and undefined cell populations. These “mystery” cell populations are very likely to give new insights in studying the hPSC-CM maturation process. Moreover, as shown by Biendarra-Tiegs et al., a single parameter is likely not enough to fully characterize the state of a cell. By combining various single-cell resolution assays, researchers are likely to gain a more comprehensive look of the state of a cell. Furthermore, the heterogeneity observed in these studies accentuate the demands for establishing maturation metrics to address hPSC-CM heterogeneity.

## 6. Future perspectives

In this review, we summarized key changes in the CM maturation process, transcriptomic studies looking at major gene regulatory networks and pathways in CM maturation, and recent efforts in studying hPSC-CMs in vitro using various single-cell resolution assays. The majority of hPSC-CMs generated in vitro are still immature and fail to recapitulate adult CMs in morphology, functionality, and transcriptome. The field is, therefore, intensively focusing on developing new strategies to improve PSC-CM maturation for better cardiac disease modeling, drug and toxicity testing, and regenerative medicine. In this regard, understanding the molecular, cellular, and developmental mechanisms underlying this process will be essential and instrumental for generating mature PSC-CMs in a dish. Given that a number of PSC-CM maturation procedures are currently developed and employed by research laboratories, it would be also crucial to establish standardized platforms to index maturation status quantitatively and address contradictory data, resulting from differences in cell culture protocols, tissue engineering platforms, and inherent differences among hPSC lines.

Single-cell resolution assays have now been increasingly developed and utilized for biomedical research. In particular, high-quality scRNA-seq enables building developmental trajectories of CNs undergoing maturation and identifying putative factors mediating the process. Thus, scRNA-seq technology is expected to help us gain a deeper understanding of PSC-CM maturation. scRNA-seq-based maturation metrics are also expected to hasten the translation of hPSC-CMs to the clinic as researchers could directly compare different strategies to promote maturation. This will help identifying strengths and weaknesses of the existing protocols and selecting the most mature hPSC-CMs for more rationale disease modeling and pharmacological testing.

## Figures and Tables

**Table 1:** PSC-CM maturation studies at single-cell resolution. (Abbreviations: ACDY5, CM-specific adenylyl cyclase isoform; ACTN2, alpha-actinin 2; AP, action potential; APD, action potential duration; CACNA, calcium voltage-gated channel; CALM2, calmodulin 2; CASQ2, calsequestrin 2; Cx43, connexin 43; DES, desmin; FGF12, fibroblast growth factor 12; IHL1, four and a half LIM domains 1; GJA1, gap junction protein alpha 1; HOPX, homeodomain-only protein; JUN, Jun proto-oncogene, AP-1 transcription factor subunit; KCNA4, potassium voltage-gated channel subfamily A member 4; KCND3, potassium voltage-gated channel subfamily D member 3; KCNJ3, potassium inwardly rectifying channel subfamily J member 3; LDHA, lactate dehydrogenase A; LDHB, lactate dehydrogenase B; MLC, myosin light chain; MYH, myosin heavy chain; MYL, myosin light chain; NPPA, natriuretic peptide A; NPPB, natriuretic peptide B; PDLIM3, PDZ and LIM domain protein 3; PITX2, paired like homeodomain 2; PLN, phopholamban; PPARGC1A, PPARG coactivator 1 alpha; RMP, resting membrane potential; RV, right ventricle; SLMAP, sarcolemma associated protein; TBX3, T-box transcription factor 3; TCAP, telethonin; TNN, troponin; TRDN, triadin; Vmax, maximum upstroke velocity.).

Reference	Methods	Transcriptomics	Electrophysiology	Proteomics	Single-cell evaluation method	Timepoints for single-cell analysis
Giacomelli et al. [113]	3D microtissue co-culture with cardiac fibroblasts (CFs) and cardiac endothelial cells (CEs)	Sarcomeric genes: TNNT2, TNNI1, TNNI3 ↑ MYL2, MYL3, MYL4 ↑ DES, TCAP ↑ Potassium channel protein genes: KCND3, KCNA4 ↑ Ca^2+^ handling protein genes: CASQ2, CALM2, PLN, TRDN ↑ Metabolic genes: LDHA ↓ LDHB ↑ Adrenergic signaling pathway protein gene: ACDY5 ↑ Gap junction protein gene: GJA1 (Cx43) ↑	N/A	N/A	Single-cell RNAseq	Not specified, likely Day 21 post microtissue construction
Lam et al. [114]	Human cardiac tissue strip (hCTS)	Cardiac development gene: NPPB ↑ RV development gene: PDLIM3 ↑ Calcium handling genes: TNN13, JUN, PLN ↑	N/A	N/A	Single-cell RNAseq	Not specified, likely Day 14 post microtissue construction
Lam et al. [114]	Human cardiac anisotropic (hCAS)	Sarcomeric genes: MYL2, MYH7, TNNI3 ↑ MYH6, TNNI1 ↓ Cardiac development genes: HOPX, NPPA, NPPB ↑ RV development genes: PDLIM3, ACTN2 ↑	N/A	N/A	Single-cell RNAseq	Not specified, likely Day 14 post microtissue construction
Biendarra-Tiegs et al. [115]	Prolonged culture (~6 weeks)	Ion channel regulator genes: SLMAP, FGF12, FHL1 ↑ Potassium channel KCNJ3 ↑ L-type calcium channel gene: CACNA2D1 ↑ Sarcomeric gene: MYL2 ↑ Transcriptional factor genes: TBX3, PITX2 ↓	AP amplitude ↑ Vmax ↑ Mean APD_90_/APD_50_ ↑ Sensitivity to tetrodotoxin (sodium channel blocker) ↑ Population with atrial-like AP (APD_90_/APD_50_ >1.4) emerged after D30	N/A	Single-cell RNAseq ArcLight electrophysiology analysis	Day 12, 40 in differentiation
Jabart et al. [116]	Matrigel coated 6-well cell culture plates	N/A	N/A	MLC2V ↑ (almost threefold) (largest increase in MLC2A & MLC2V co-expressing cells) MLC2A ↑ (marginally)	Single-cell westerns	Day 17, 23, 30 in differentiation
